# Putting nutrition education on the table: development of a curriculum to meet future doctors’ needs

**DOI:** 10.1017/S0007114522001635

**Published:** 2023-03-28

**Authors:** Glenys Jones, Elaine Macaninch, Duane D. Mellor, Ayela Spiro, Kathy Martyn, Thomas Butler, Alice Johnson, J. Bernadette Moore

**Affiliations:** 1 Association for Nutrition, London, UK; 2 Education and Research in Medical Education (ERimNN) Brighton and Sussex Medical School, Brighton, UK; 3 NNEdPro Global Centre for Nutrition and Health, Cambridge, UK; 4 Aston Medical School, Aston University, Birmingham, UK; 5 British Nutrition Foundation, London, UK; 6 Education and Research in Medical Education (ERimNN), School of Sport and Health Sciences, University of Brighton, Brighton, UK; 7 Faculty of Health, Social Care and Medicine, Edge Hill University, Ormskirk, UK; 8 School of Food Science and Nutrition, University of Leeds, Leeds, UK; 9 The Nutrition Society, London, UK

**Keywords:** Nutrition education, Medical doctors, Medical students, Curriculum, Obesity, Malnutrition, Integrated person-centred care

## Abstract

COVID-19 has further exacerbated trends of widening health inequalities in the UK. Shockingly, the number of years of life lived in general good health differs by over 18 years between the most and least deprived areas of England. Poor diets and obesity are established major risk factors for chronic cardiometabolic diseases and cancer, as well as severe COVID-19. For doctors to provide the best care to their patients, there is an urgent need to improve nutrition education in undergraduate medical school training.

With this imperative, the Association for Nutrition established an Interprofessional Working Group on Medical Education (AfN IPG) to develop a new, modern undergraduate nutrition curriculum for medical doctors. The AfN IPG brought together expertise from nutrition, dietetic and medical professionals, representing the National Health Service (NHS), royal colleges, medical schools and universities, government public health departments, learned societies, medical students, and nutrition educators. The curriculum was developed with the key objective of being implementable through integration with the current undergraduate training of medical doctors.

Through an iterative and transparent consultative process, thirteen key nutritional competencies, to be achieved through mastery of eleven graduation fundamentals, were established. The curriculum to facilitate the achievement of these key competencies is divided into eight topic areas, each underpinned by a learning objective statement and teaching points detailing the knowledge and skills development required. The teaching points can be achieved through clinical teaching and a combination of facilitated learning activities and practical skill acquisition. Therefore, the nutrition curriculum enables mastery of these nutritional competencies in a way that will complement and strengthen medical students’ achievement of the General Medical Council (GMC) Outcome for Graduates.

As nutrition is an integrative science, the AfN IPG recommends that the curriculum is incorporated into initial undergraduate medical studies before specialist training. This will enable our future doctors to recognise how nutrition is related to multiple aspects of their training, from physiological systems to patient-centred care, and acquire a broad, inclusive understanding of health and disease. In addition, it will facilitate medical schools to embed nutrition learning opportunities within the core medical training, without the need to add in a large number of new components to an already crowded programme or with additional burden for teaching staff.

The undergraduate nutrition curriculum for medical doctors is designed to support medical schools to create future doctors who will understand and recognise the role of nutrition in health. Moreover, it will equip frontline staff to feel empowered to raise nutrition-related issues with their patients as a fundamental part of enhanced care and to appropriately refer on for nutrition support with a registered associate nutritionist/registered nutritionist (ANutr/RNutr) or registered dietitian (RD) where this is likely to be beneficial.

The relationship between malnutrition (which includes undernutrition, overnutrition and micronutrient deficiencies) and ill health is unequivocal^(1)^. Globally suboptimal diets are now the leading preventable cause of morbidity and mortality from a range of chronic diseases, in particular CVD, cancers and type 2 diabetes^(2)^. Specifically, in 2017, inadequate dietary patterns (high in Na; low in wholegrains, fruit, vegetables, nuts and seeds, and *n*-3 fatty acids) were responsible for more deaths globally than tobacco smoking. Notably however, evidence suggests that making dietary changes in line with those of public health recommendations across the globe (e.g. increased wholegrains, fish, vegetables and decreased processed meats, sugar and salt) may be associated with increases in life expectancy^(3)^. Modelling of data from the Global Burden of Disease Study has suggested sustained dietary change, from a Western diet to an optimised healthy diet made at the age of 20 years and upheld through life, could increase life expectancy by over a decade^(4)^. Although such modelling studies have their caveats, these data are in line with numerous population studies that show following healthier dietary practices to be correlated with reductions in mortality risk from chronic diseases such as hypertension, obesity, diabetes, CVD and some cancers^(3,5–7)^.

Undernutrition is a poorly recognised public health problem, associated with many adverse outcomes including increased hospital admissions, longer lengths of hospital stay clinical complications and increased mortality^(8)^. In the UK, prior to the pandemic, undernutrition was estimated to affect over 3 million people at a cost of £23·5 billion^(8)^. Simultaneously, the prevalence of overweight and obesity in the UK has never been higher and, in England alone, costs the NHS £6·1 billion and wider society £27 billion annually^(9)^. Almost two-thirds of English adults (63 %; 67 % of men and 60 % of women) are overweight (a BMI ≥ 25 kgm^–2^) and more than one in four (27 %; 26 % of men and 29 % of women) are living with obesity (a BMI ≥ 30 kgm^–2^)^(10)^. New data from the National Child Measurement Programme in England suggests a dramatic increase in both the percentage of children at reception (ages 4–5 years) and year 6 (ages 11–12 years) living with overweight and obesity^(11)^. Moreover, the deprivation gap has widened. For instance, at reception the prevalence of obesity is 20·3 % in children from the most deprived families, in contrast to 7·8 % in children from the least deprived families^(11)^.

While excess weight has been a major focus of public health, it is important to recognise the significance of micronutrient deficiencies arising from poor-quality diets and suboptimal dietary patterns. UK dietary survey data suggest that substantial proportions of some population groups have low intakes of various essential vitamins and minerals^(12,13)^, and income analysis suggests that intakes of most micronutrients tend to increase with income^(14)^. The pandemic has shone harsh light on persistent and widening health, socio-economic and geographical inequalities, as well as inequality in the prevention, management and treatment of malnutrition^(15,16)^. For example, in a retrospective longitudinal study exploring UK and US pandemic diet and lifestyle behaviours, disruption of healthy lifestyle behaviours was higher in younger, female and socio-economically deprived participants^(17)^. Separately, NHS Digital data showed an almost 50 % increase in the number of under-20s admitted to hospital with an eating disorder in 2020–21 compared with the previous year, with the number exceeding 3200^(18)^. This was reported to be a result of insufficient community support provision to meet demand, leading to an increase in the number reaching the point of hospitalisation^(18)^.

Adults aged over 60 years are more likely than younger age groups to suffer from malnutrition. A recent Age UK survey reported that since the beginning of the pandemic, 1·4 million older people in England aged 60+ years have been eating less and 3·7 million reported that they, or others in their household, have been unable to eat nutritious food. Therefore, pandemic-related stressors have further increased the risk of undernutrition and malnutrition in this already vulnerable age group^(19)^. Socio-economic challenges that decrease food access, including food poverty and food insecurity, negatively impact on physical and mental health and quality of life and underscore the complex biological, social and environmental determinants of malnutrition^(16,20,21)^. This has been recognised in public health policy and white papers such as the National Food Strategy and Levelling Up the UK^(22)^. The National Food Strategy aimed to help widen focus on the interplay between food and health systems, climate change, and economic and political drivers^(23)^. The combination of stressors (COVID-19 and obesity pandemics as well as climate change) perhaps means that the need for effective and evidence-based nutrition education for medical doctors and healthcare professionals has never been so critical.

The specific role of medical doctors in addressing nutrition in clinical practice has been acknowledged by multiple authoritative professional bodies^(24)^. The GMC’s Outcomes for Graduates sets out the following for what newly qualified doctors must be able to do with respect to nutrition: recognise where poor nutrition is contributing to ill health; take action by seeking advice from colleagues and making appropriate referrals; apply principles and knowledge relating to nutrition to medical practice and integrate these into patient care; and lastly, discuss the role and impact of nutrition to the health of individual patients and societies^(25)^. Moreover, in the 2019 NHS Long Term Plan, which has a major focus on the prevention of disease and health inequalities, a commitment was set to ensure nutrition has a greater place in professional education training. This was specific, so that doctors would be encouraged to address the role of nutrition in health in an informed and sensitive way and refer cases appropriately where nutrition support is required^(26)^.

Intuitively, equipping the next generation of medical doctors with appropriate nutrition competencies should support disease prevention and improve clinical outcomes^(27,28)^. Yet while medical students and trainees acknowledge they need to develop their skills and knowledge in nutrition, there are widespread reports of insufficient nutrition education during medical training in UK and globally^(27,29–32)^. Pooled survey and evaluation data suggest most UK medical students and doctors feel their nutrition training was inadequate, with more than 70 % reporting they could identify less than 2 h across their academic and clinical training^(29)^. Separate research has suggested that in fact students underestimate the amount of nutrition content in their medical education^(30,33,34)^. This documented divergence between medical students’ perception of nutrition content and actual teaching hours perhaps highlights a need for nutrition teaching to be explicitly flagged to students^(30,33,34)^.

This will inevitably not be without challenges. How to best incorporate nutrition knowledge and clinical skills as components of medical education and clinical practice in an already complex and demanding medical curriculum may require new integrative approaches^(35)^. However, tackling these challenges is critical to provide the effective nutrition care required given the current levels of malnutrition and chronic disease patterns. Doctors do not need to be nutritionists, but they can play a critical role in reducing the health impacts of poor nutrition. Namely, by recognising the contribution of nutrition in clinical and population health, and developing the knowledge, skills and confidence to either offer advice or refer on appropriately depending on the context^(36)^. Knowledge and practice in appropriate referrals to specialists are important, because if doctors or healthcare professionals are not providing nutrition information and advice, patients will seek information elsewhere. Evidence-based nutrition and accurate information on food and health can be difficult to find in a contested space filled with commercial interests, social media and influencers^(36,37)^.

## A nutrition curriculum for nutrition competency standards

Responsibility for the undergraduate nutrition curriculum for medical students was transferred from the Academy of Medical Royal Colleges (AoMRC) to the Association for Nutrition (AfN) in 2018. Founded in 2008, the AfN is a charity and the independent regulator that accredits university undergraduate programmes awarding undergraduate and taught postgraduate (i.e. BSc and MSc) degrees in Human Nutrition in the UK. In addition to providing quality assurance schemes for the assessment of nutrition training, AfN is responsible for assessing the professional competency of nutritionists and awarding the professional titles of registered nutritionist (RNutr) and registered associate nutritionist (ANutr)^(38)^.

While a working group of the AoMRC had previously outlined what newly qualified doctors should understand about nutrition^(39)^, this guidance was not embedded in competency standards. Such standards can help to determine the required knowledge and skills for safe and effective care aligned to optimise health along with patients’ priorities^(40)^. In addition, competency standards can provide a useful framework to support curriculum developers within medical schools to provide nutrition training that is relevant for both clinical medical practice and management of student expectations. With transfer of responsibility for the undergraduate nutrition curriculum for medical students to the AfN, the AoMRC and GMC gave their support for AfN to develop a modern curriculum that supports the achievement of GMC outcomes for medical graduates and provides the fundamental nutrition knowledge and skills needed by our future doctors^(38)^.

## Methods

To understand how this should be best achieved, and to ensure realistic deliverability of the updated curriculum into core teaching for medical students, the AfN formed an Interprofessional Working Group on Medical Education (AfN IPG). The group brought together expert professionals and organisations, as well as those who would play a key role in delivery of, or be influenced by, the updated curriculum. The working group represented Public Health England, NHS England, nutrition and dietetic professionals, medical royal colleges, medical schools, medical students, doctors and training providers. The nutrition curriculum was developed through collaborative and open discussion between group members over a number of meetings, with an agreement reached by consensus over the required detail and structure. It was developed without external funding, with all parties freely donating their time and expertise. Any declarations of interests were required to be reported by working group members at each meeting.

Both the current requirements and multiple additional opportunities that exist within the GMC’s Outcomes for Graduates to include nutrition in their core curriculum for undergraduate medical training are outlined in Table 1. These demonstrate the key roles that nutrition plays across the central GMC themes and provide a strong rationale for the development of an integrated curriculum, whereby nutrition is taught within core and speciality training, and not as a separate stand-alone topic. Indeed, it is an integrated curriculum where, throughout students training, the relationship between nutrition, health and disease is reiterated as fundamental and essential for optimal patient-centred care.

**Table 1.** Identification of where the requirement or opportunity exists to include nutrition within the expected curriculum for medical students in the UK, as defined by the UK General Medical Council’s Outcomes for Graduates^(25)^

GMC themes	Nutrition is a required learning outcome	Nutrition not explicit but essential for good patient care	Nutrition could be used to enhance learning and achieve learning outcomes
Professional values and behaviours: This could be applied to five of six outcome domains	Safeguarding vulnerable patients: This includes recognition of poor nutrition and taking action or referral as appropriate	Dealing with complexity and uncertainty: Nutrition should be a part of considering patient’s goals and priorities for enhanced care	Professional and ethical responsibilities: Both for self-care and reflection on student/doctor’s own lifestyle, attitudes and beliefs; and how these might impact advice given Patient safety and quality improvement: Food and nutrition projects could be used to achieve many of the learning outcomes in this domain Leadership and team working: This includes interdisciplinary working across healthcare settings that will have food and nutrition as part of their services
Professional skills: This could be applied to two out of four outcome domains		Prescribing medications safely: Route of administration, timing and interactions with food and dietary supplements essential for safe and effective care	Communication and interpersonal skills: Conversations about role of nutrition in health can be used to introduce these skills
Professional knowledge: This could be applied to five out of six outcome domains	Applying biomedical scientific principles: This explicitly mentions nutrition and links it both to the science and practice of medicine	Applying psychological principles: The relationship of psychological and medical conditions and impact of behaviour on treatment both are impacted by diet and nutrition	Clinical research and scholarship: Nutrition-focused examples can be useful as teaching material for statistics, research design and use of evidence
	Health promotion and illness prevention: This domain specifically mentions healthy weight and diet along with the role and impact of nutrition on health	Applying social science principles: Nutrition provides both good teaching examples and requires the application of social science principles to be effective in practice	

**Fig. 1.**
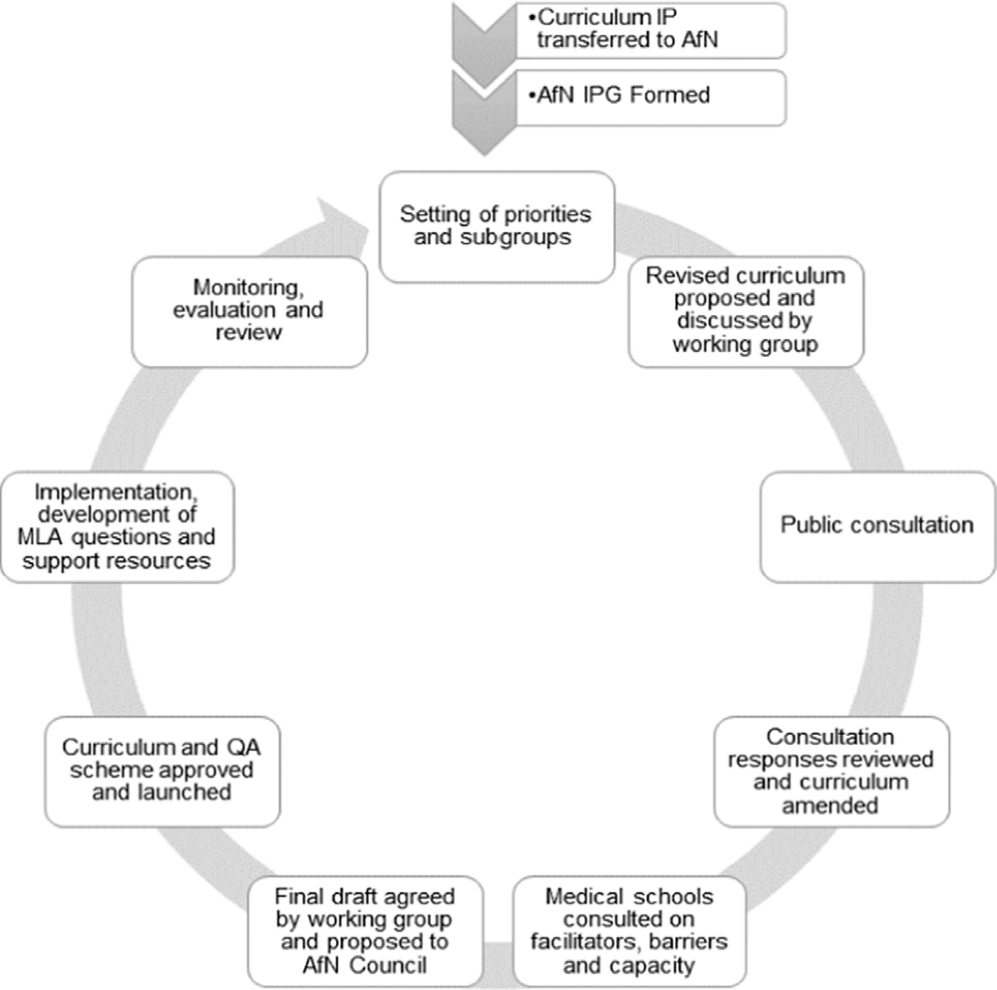
Flow chart of the development steps in creating the nutrition curriculum for undergraduate medical education. MLA, medical licensing assessment.

In September 2020, a public stakeholder consultation was held on an initial draft curriculum document. This gave a wide body of stakeholders (medical schools, royal colleges, medical and nutrition organisations, training providers, nutrition and dietetic students, medical professionals and nutrition professionals) the opportunity to provide feedback on the proposed curriculum. Targeted questions encouraged comments on the curriculum structure, content and achievability. In addition, stakeholders in medical schools were invited to comment on their local practices. Namely, details on local nutrition expertise and teaching practices, and examples of nutrition inclusion within current core teaching in respondent’s medical schools, were solicited. Stakeholders were also asked to comment on what they viewed as potential implementation barriers as well as facilitative opportunities. The working group reviewed the responses to the consultation to produce the final version of the undergraduate nutrition curriculum, alongside the identification of activities that would be beneficial to support its implementation such as the production of suitable teaching resources and capacity assessment. The iterative cycle used for the development of the curriculum and its continual evaluation is illustrated in Fig. 1.

### The Association for Nutrition undergraduate nutrition curriculum for medical students

**Fig. 2.**
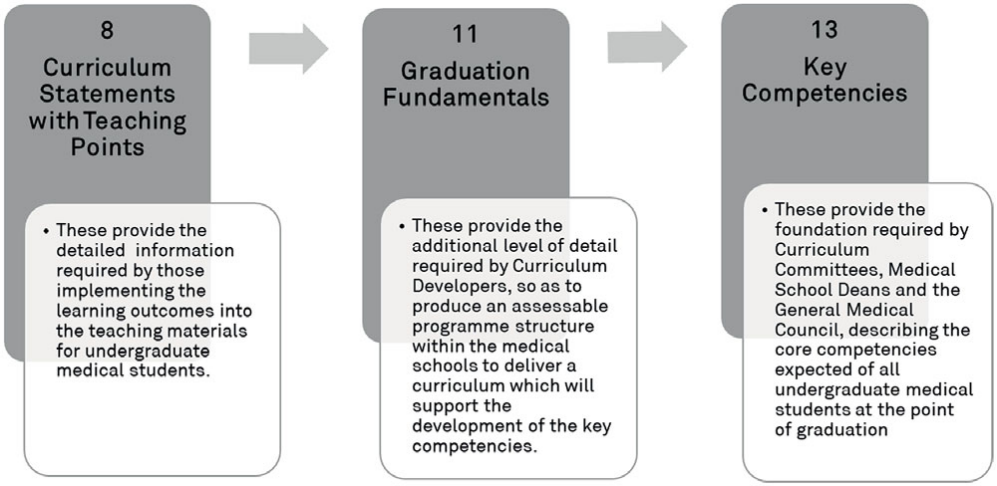
Key components of the nutrition curriculum for undergraduate medical education.

The AfN Undergraduate Curriculum in Nutrition for Medical Doctors^(41)^ has been designed to be presented to medical students as an integral part of their general undergraduate training, making it clear how nutrition interrelates with the study of other systems and contributes to an inclusive understanding of health and disease. The structure of the curriculum document is outlined in Fig. 2. This illustrates how the curriculum statements with teaching points will support undergraduate medical students through the achievement of eleven graduation fundamentals to develop thirteen core nutritional competencies by the point of graduation.

**Fig. 3.**
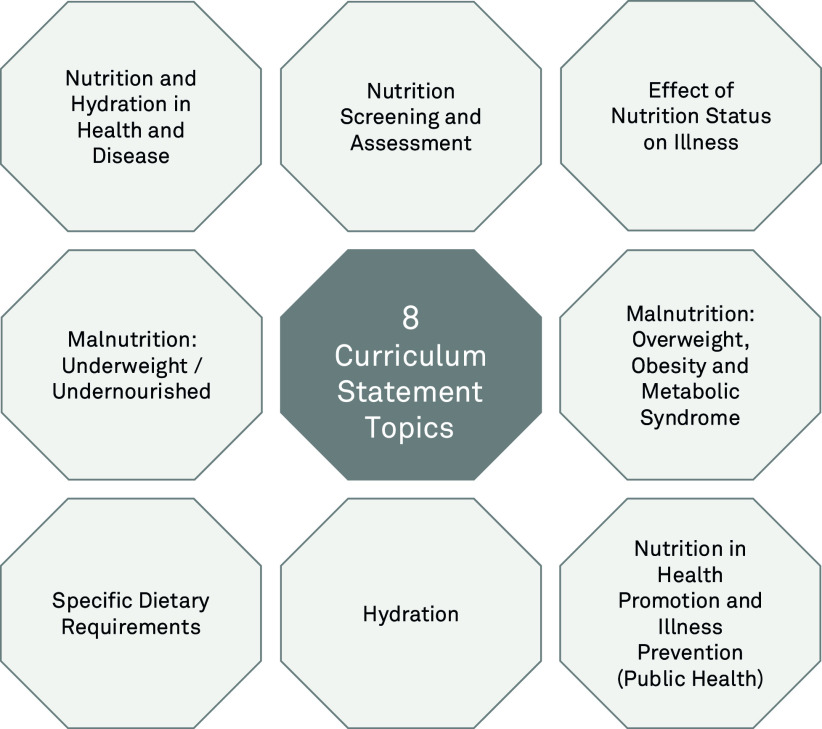
Diagram of the eight topic areas the curriculum and teaching points address.

The curriculum statements and teaching points build knowledge and understanding in eight critical nutrition topic areas outlined in Fig. 3. Each topic is accompanied by a statement of the learning objective and detailed teaching points to support students acquisition of the key competencies. The teaching points can be achieved through a combination of facilitated learning activities, such as lectures, case-, team- or problem-based learning, supporting resources and activities and practical skills acquisition through clinical teaching, both simulated and at the ‘bedside’, thereby providing sufficient opportunity for students to meet all the learning outcomes detailed in this curriculum and achieve the key competencies for graduates in a way that complements their achievement of the GMC Outcome for Graduates^(25)^.

### Support activities

To support the implementation of nutrition teaching, the curriculum has been mapped to facilitate the achievement of the GMC’s Outcomes for Graduates^(25)^ at the level of key competencies, graduate fundamentals and curriculum statements. In addition, it has been identified where in the domains of the new medical licensing assessment (MLA), due to commence in 2024^(41)^, nutritional factors can either be a causal or influencing factor, or be impacted by the clinical condition or treatment.

The AfN also hosts a resource support section on their website^(42)^, containing case studies from medical schools that have successfully incorporated nutrition training into their core teaching. These demonstrate how programmes have approached embedding nutrition within their curriculum and provide examples other programmes can use to aid curriculum reviews. To aid identification of suitable resources to support the delivery of the curriculum’s teaching points, the AfN has introduced a quality assurance scheme for resources to be assured as evidence-based and suitable for delivery of specified teaching points. The AfN IPG will also develop a bank of suitable nutrition questions for inclusion in the MLA and will submit these to the GMC, with amendments and new questions submitted as appropriate.

## Discussion

In summary, a consensus-led, multi-stakeholder process led by the AfN has developed a modern nutrition curriculum for undergraduate medical students in the UK. The AfN IPG recommends that medical schools deliver thirteen core nutritional competencies in order for future medical doctors to master eleven graduation fundamentals in nutrition by the point of their graduation^(41)^. The strengths of the AfN IPG process included as follows: the involvement of a multi-stakeholder, expert group; the fundamental consideration of practical application and implementation of the curriculum by medical schools; the iterative feedback from medical schools throughout; and an expansive public consultation process. The widespread adoption of this curriculum will provide consistency for the expectations and requirements for nutrition education in a manner that fully complements the GMC’s Learning Outcomes for Graduates^(25)^. Therefore, the key teaching points and graduate fundamentals outlined in this new nutrition curriculum should be linked to the MLA.

### Opportunities for implementation

A potential challenge raised by medical schools will be how to incorporate, what to some may first appear to be additional material, nutrition into an already incredibly dense curriculum^(24,29)^. Clearly signposting to where nutritional science already exists in undergraduate medical foundational training is a clear first step that medical schools should take. For example, the core underpinning foundation of medical training, for example biochemistry and physiology, can be presented through both a medical and nutritional science lens. Fundamentally, cellular metabolism and organ physiology, both in human health and disease, are completely interdependent on the catabolism and anabolism of substrates derived originally from dietary nutrients (and therefore food). Complementing this, the considered appraisal of nutrition status during history taking and person-centred approaches to patient assessment^(39,43)^ should be presented as requisite skills to enhance patient engagement.

The key is to explicitly highlight nutrition content where it is being taught, so medical students know it is core to their learning and can develop nutritional competencies relevant to medical practice as they progress. Rather than presenting nutrition as a stand-alone subject, it should be integrated into a holistic model of care. For example, nutrition is an important consideration in safe prescribing, to prevent drug nutrient interactions^(44)^, as well as an important non-pharmacological treatment that can reduce polypharmacy where appropriate^(45–48)^. Although medical schools adhere to the same rigorous consistent standards for graduate expectations, curriculum design between schools varies from more traditional models (e.g. pre-clinical science teaching in early years followed by clinical teaching later) to more integrated models that have clinical placements starting from year 1^(49)^. This underscores that a variety of approaches to nutrition education are required.

Indeed, the new curriculum offers an incentive for medical schools to benchmark expected nutrition education outcomes, recognised as a crucial first step to improve nutrition education implementation^(40)^. This provides curriculum developers the opportunity to map the nutrition that is currently taught in their programmes and further integrate nutrition where appropriate as a critical element of clinical assessment and treatment. Moreover, as highlighted in Table 1, a critical review of the GMC’s current Outcomes for Graduates^(25)^ shows that the majority of learning domains either include nutrition explicitly or provide opportunity to enhance the learning of medical students through the inclusion of nutritional content and examples of nutritional practice in the provision of optimal healthcare. Emphasising the role of nutrition in these learning domains adds weight to the core nature of nutrition in medical practice.

Once teaching and materials have been embedded within the curricula, then formal and rigorous assessment of taught content needs to follow, and therefore be developed, as assessment can help to focus and support student learning^(50)^. Assessment of nutrition should be developed for the early and later years of Objective Structured Clinical Examination (OSCE) and incorporated into applied knowledge assessments like the Prescribing Safety Assessment (PSA), as well as into the MLA and postgraduate examinations. Nutrition can be integrated into complex cases to aid decision-making in situational judgement tests, which are used as a part of the selection process for employment onto foundation programmes post-graduation^(51)^. This will require nutrition-trained faculty to be involved in the development of these assessments, which are ultimately needed to provide authentic assessment and produce doctors who are competent to use nutrition as a therapeutic option on graduation and throughout their careers.

### Challenges

The place of nutrition in medical curricula has been reported as being peripheral^(52)^. Often nutrition may be included as co-curricular or extracurricular activities, such as student selected components, elective courses, research projects or external opportunities through student society-led groups such as Nutritank^(53)^. Currently, medical doctors report rarely including nutrition in clinical care with various cited reasons including time and confidence^(29)^. In part, this is likely due to a lack of nutrition education in their own training^(41)^.

Clinical role models are a key element of professional development recognised as an important hidden curriculum in healthcare professional training^(49)^. Accessing appropriately qualified nutrition teachers from within the core medical teaching establishment can be problematic, necessitating a more interfaculty or even interinstitutional teaching model^(49,54)^. A lack of professional role models in placements and faculty trained in nutrition is a clear barrier to adequate nutrition education in medical schools and to demonstrating how nutrition is integrated into clinical practice^(55)^. Making better use of allied health professionals such as ANutr/RNutr, registered dietitians (RD) and nutrition-trained nurses and pharmacists in multidisciplinary teams during clinical and community training offers the opportunity to enhance both interprofessional skills and the nutrition knowledge of future medical doctors.

In some universities, there may be an opportunity to collaborate with either nutrition course faculty, ANutr/RNutr or RD, but these may be limited by availability and capacity. Although interprofessional education is becoming more prevalent in medical schools, some dietitians still report facing challenges in influencing the medical curriculum^(56)^. This alongside discipline differences in approaches to education may also mean nutrition and dietetic professionals would need to adapt their approach and style of delivery to match that expected on medical degrees. Structurally and culturally, medical education may historically have been distinct in its specialism approach, context and assessment, relative to other health professions. However, the drive across all healthcare education to incorporate interprofessional education provides an opportunity to explore nutrition across professional domains and challenging restrictive action of healthcare professionals being predominately taught by their own profession^(57)^.

Many of the key aspects of the new nutrition curriculum support interprofessional education as a common thread for all health professionals as they explore patient-focused case studies through the lens of their developing professional roles. This is an approach also recognised in the recently published recommendations from the UK Obesity Care Competencies Working Group facilitated by the College of Contemporary Health^(58)^. These timely *obesity care competencies for healthcare education in the UK* ^(58)^ are complementary to the nutrition curriculum, and ideally both sets of competencies should be mapped and adopted by medical schools in tandem. Indeed, more collaboration to develop the right interprofessional education content and activities and facilitate medical teaching capacity is needed. Opportunities exist for medical schools to collaborate with local universities and organisations such as the AfN, the British Dietetic Association (BDA), the Nutrition Society, and the NNEdPro Global Centre for Nutrition and Health. In addition, there are clear opportunities to incorporate and utilise resources produced by nutrition experts from the British Association for Parenteral and Enteral Nutrition (BAPEN), the World Cancer Research Fund (WCRF) and Health Education England, among others.

Interest in nutrition will likely not be universal among medical students and faculty^(59)^. Although some groups, including those led by students such as Nutritank, have pushed for incorporating more nutrition training, others might not see nutrition as a priority to the immediate needs of patients on initial medical consultation. Indeed, in a European survey of medical school faculty, those responding did not feel more nutrition education was required^(34)^. This may reflect the traditional pharmacological and surgical approaches to medical treatments and may be more apparent in some specialities than others, for example, in acute medicine and surgery where medical priorities may represent life or death decisions. Therefore, incorporating diversity within faculties of medical educators, to include those from other health and nutrition professions, will likely facilitate the integration of nutrition into local programmes in the first instance. Once integrated, mapping learning to national graduate expectations and high-stake examinations will ensure the presence of nutrition in the curriculum of local medical schools beyond the interests of championing faculty.

### Conclusions

Nutrition is a key modifiable factor in the prevention of disease and healthy ageing. Given the current extraordinary prevalence of diet-related chronic disease in the UK, and the integral role nutrition plays in the treatment and rehabilitation of disease, it is now imperative that nutrition fundamentals be embedded in core undergraduate training for medical doctors and be assessed in the new MLA in 2024. Medical doctors do not need to become nutritionists or dietitians but should be equipped to confidently address malnutrition in all its forms. Doctors who will see thousands of patients throughout their career play a key role in helping to treat and monitor nutrition-related conditions, as well as in delivering preventative medicine. Future doctors should therefore be skilled to discuss factors such as achieving a healthy weight in an informed and sensitive manner, as well as having the knowledge to refer patients to further nutrition support when appropriate. There is a clear opportunity now for medical schools to distinguish themselves based on the integration of nutrition practice into holistic healthcare training to adequately prepare graduates with the knowledge and skills in nutrition care with the ultimate goal of improving patient care.
